# Incidence and Related Factors for Low-Extremity Deep Vein Thrombosis in Breast Cancer Patients Who Underwent Surgical Resection: What Do We Know and What Should We Care

**DOI:** 10.3389/fsurg.2022.755671

**Published:** 2022-02-04

**Authors:** Liqiang Chen, Qiang Feng, Wenjuan Wang, Lanbo Liu

**Affiliations:** ^1^Department of Cardiovascular, the Fourth Hospital of Hebei Medical University, Shijiazhuang, China; ^2^Department of Cardiovascular, Handan Central Hospital, Handan, China; ^3^Department of Emergency, 982 Hospital of the Joint Logistics Support Force of The Chinese People's Liberation Army, Tangshan, China

**Keywords:** risk factor, incidence, predict model, low-extremity deep vein thrombosis, breast cancer

## Abstract

Malignancy, surgical resection, and neoadjuvant and/or adjuvant chemotherapy increase the low-extremity deep vein thrombosis (LDVT) risk in patients with breast cancer, bringing in great physical burdens, disabilities, and worse survivals. However, LDVT in surgical breast cancer patients is scarcely reported. Therefore, this study aimed to evaluate the incidence and related factors for LDVT in these patients. Patients with breast cancer who underwent surgical resection were included. LDVT was examined on the day of discharge and 1 month after the discharge. A total of 491 eligible patients were included, among which 11 (2.2%) patients occurred LDVT. Besides, higher age, history of diabetes mellitus, advanced T and tumor node metastasis (TNM) stages, higher platelet count, and shorter activated partial thromboplastin time (APTT) were correlated with increased LDVT incidence (all *p* < 0.05). Additionally, higher age [*p* = 0.004, odds ratio (OR) (95% CI): 1.082 (1.023–1.144)], history of diabetes mellitus [*p* = 0.003, OR (95% CI): 10.426 (2.219–48.986)], and a higher platelet count [*p* = 0.008, OR (95% CI): 1.017 (1.004–1.029)] were independent factors for increased LDVT incidence, while higher APTT [*p* = 0.004, OR (95% CI): 0.636 (0.467–0.866)] was an independent factor for decreased LDVT incidence. Lastly, the risk prediction model involving age, history of diabetes mellitus, platelet count, and APTT showed a good ability to predict LDVT occurrence (area under curve: 0.919, 95% CI: 0.869–0.968). In conclusion, the LDVT incidence is 2.2%, and its independent factors consist of age, history of diabetes mellitus, platelet count, and APTT in patients with breast cancer who underwent surgical resection, which provides evidence for the prevention and surveillance of LDVT in surgical breast cancer.

## Introduction

Breast cancer is the most common carcinoma and a leading cause of cancer mortality in women, with ~2,000,000 new cases and 626,000 fatalities globally every year (1, 2). So far, great progress has been made on the treatments for breast cancer, such as surgical resection, neoadjuvant therapy, adjuvant therapy, endocrinotherapy, and targeted therapy (3–7). Among these treatments, resection is a predominant strategy. Unfortunately, patients with breast cancer after resection may experience severe postoperative complications (8–11). One of the most threatening complications is deep vein thrombosis (DVT), a severe disease in the venous system, which is a vital cause of high mortality of breast cancer (10, 12, 13).

Among DVT, low-extremity DVT (LDVT) occurs more frequently because the venous blood viscosity of low-extremity is obviously higher than that of upper-extremity (14). According to previous studies, the risk factors of LDVT include major surgery, malignant tumor, older age, diabetes mellitus, etc. (15–19). Meanwhile, it is worth noting that patients with LDVT often suffer from physical burdens, such as Neuh of positive and Homans signs, superficial varicosities, stasis pigmentation, edema and pain of lower limbs, and low-extremity paralysis, resulting in treatment difficulty, reduced quality of life, worse survivals, or sudden death (9, 20–25).

The common LDVT incidence is low in patients with breast cancer (26), but this incidence risk is higher for those who received surgical resection, neoadjuvant, and/or adjuvant chemotherapy (27, 28). Therefore, great attention should still be attached to LDVT in surgical breast cancer patients. However, few such studies have been reported on this.

Therefore, this study aimed to evaluate the incidence of LDVT and its related factors in surgical breast cancer patients.

## Materials and Methods

### Patients

After approval by the Institutional Review Board of The Fourth Hospital of Hebei Medical University, a total of 491 patients with breast cancer who underwent surgical resection in our hospital from January 2016 to September 2020 were included consecutively in this study. The inclusion criteria were as follows: (i) diagnosed as primary breast cancer by the histopathological examination; (ii) scheduled for surgical resection; (iii) aged older than 18 years; and (iv) able to understand the study contents and willing to participate in the study. The exclusion criteria were as follows: (i) presented with thrombosis before surgery or had a history of thromboembolism; (ii) known cardiovascular diseases, cerebrovascular diseases, or hematological diseases; (iii) complicated with other cancers; (iv) received treatment with anticoagulant within 3 months before surgery; and (v) pregnant or lactating women. Written informed consents were obtained from all patients.

### Collection of Clinical Features

Clinical features of patients were documented before discharge from the hospital, which included demographic characteristics, medical history, tumor characteristics, blood routine & blood lipid indexes, and blood coagulation indexes.

### Diagnosis of LDVT

In order to avoid LDVT incidence, patients received routine perioperative low molecular weight heparin according to the guideline in China and clinicians' experience. All patients received LDVT examination on the day of discharge from the hospital and 1 month after the discharge. The diagnostic procedures of LDVT were performed as recommended in the clinical guideline (29). In brief, for patients with signs or symptoms of LDVT, a 2-level DVT Wells score was used to estimate the clinical probability of LDVT, then D-dimer test, and compression ultrasonography (gray scale, B-mode, and color Doppler) were carried out depending on the Wells score. If patients are with high clinical suspicion of LDVT but in a negative ultrasound scan, the angiography was implied to further confirm the diagnosis.

### Statistical Analysis

Data were described as count with percentage, mean with SD, or median with interquartile range (IQR). A comparison was determined by the chi-square test or the Wilcoxon rank-sum test. Univariate and multivariate logistic regression analyses were carried out to evaluate the factors affecting the LDVT occurrence and to construct the LDVT prediction model. The performance of the LDVT prediction model was estimated by the receiver operating characteristic (ROC) curve analysis. SPSS 26.0 statistical software (IBM Corp., Armonk, NY, USA) and GraphPad Prism 7.02 (GraphPad Software Inc., San Diego, CA, USA) were applied for data analysis and graph making. A *p* < 0.05 indicated statistical significance.

## Results

### Clinical Features

In the analyzed patients with breast cancer, their mean age was 52.7 ± 12.3 years, and the number of these patients with diabetes mellitus history was 47 (9.6%). Regarding the pathological grade, the number of patients with grade 1, grade 2, and grade 3 was 99 (20.2), 286 (58.2), and 106 (21.6%), respectively. With respect to the tumor size, tumor node metastasis (TNM) stage, there were 69 (14.1), 310 (63.1), and 112 (22.8%) patients with TNM stages I, II, and III, respectively. In terms of blood routine, blood lipid and blood coagulation indexes, the median (IQR) value of platelet count was 237.9 (191.6–272.9) × 10^9^/L, and the median (IQR) value of activated partial thromboplastin time (APTT) was 28.5 (27.0–30.2) s in patients. More detailed demographic characteristics, medical history, tumor characteristics, blood routine indexes, blood lipid indexes, and blood coagulation indexes in patients are presented in (Table 1).

**Table 1:** Clinical features.

**Items**	**Breast cancer patients**
	**(*N* = 491)**
**Demographic characteristics**	
Age (years), mean ± SD	52.7 ± 12.3
BMI (kg/m^2^), mean ± SD	22.8 ± 2.3
History of smoking, No. (%)	61 (12.4)
History of drinking, No. (%)	97 (19.8)
**Medical history**	
History of hypertension, No. (%)	155 (31.6)
History of hypercholesteremia, No. (%)	69 (14.1)
History of diabetes mellitus, No. (%)	47 (9.6)
**Tumor characteristics**	
**Pathological grade, No. (%)**	
Grade 1	99 (20.2)
Grade 2	286 (58.2)
Grade 3	106 (21.6)
**Molecular subtype, No. (%)**	
Luminal-A	187 (38.1)
Luminal-B	130 (26.5)
HER2^+^	83 (16.9)
Basal-like	91 (18.5)
**T stage, No. (%)**	
T1	105 (21.4)
T2	323 (65.8)
T3	63 (12.8)
**N stage, No. (%)**	
N0	276 (56.2)
N1	116 (23.6)
N2	89 (18.1)
N3	10 (2.0)
**TNM stage, No. (%)**	
I	69 (14.1)
II	310 (63.1)
III	112 (22.8)
**Blood routine and blood lipid indexes**	
WBC (x10^9^/L), median (IQR)	6.6 (5.4–7.6)
Hemoglobin (g/dL), median (IQR)	128.5 (118.4–137.3)
Platelet count (x10^9^/L), median (IQR)	237.9 (191.6–272.9)
TG (mmol/L), median (IQR)	1.2 (1.0–1.7)
TC (mmol/L), median (IQR)	4.6 (3.9–5.2)
LDL-C (mmol/L), median (IQR)	2.8 (2.4–3.3)
HDL-C (mmol/L), median (IQR)	1.5 (1.1–1.8)
**Blood coagulation indexes**	
PT (sec), median (IQR)	11.2 (9.9–12.7)
APTT (sec), median (IQR)	28.5 (27.0–30.2)
TT (sec), median (IQR)	16.4 (15.4–17.5)
FIB (g/L), median (IQR)	2.9 (2.5–3.4)
**Khorana score (or CONKO score)**	
0	432 (88.0)
1	56 (11.4)
2	3 (0.6)
**Surgery type**	
Breast conserving therapy, No. (%)	193 (39.3)
Mastectomy, No. (%)	298 (60.6)
**Length of surgery**	
Breast conserving therapy (minute), mean ± SD	88.5 ± 8.62
Mastectomy, (minute), mean ± SD	163.2 ± 15.3

*SD, standard deviation; BMI, body mass indexes; HER2^+^, Human Epidermal Growth Factor Receptor 2-positive; TNM, tumor, node, and metastasis; WBC, white blood cell; IQR, interquartile range; TG, triglyceride; TC, total cholesterol; LDL-C, low-density lipoprotein cholesterol; HDL-C, high-density lipoprotein cholesterol; PT, prothrombin time; Sec, second; APTT, activated partial thromboplastin time; TT, thrombin time; FIB, fibrinogen*.

### LDVT Incidence

**Figure 1:**
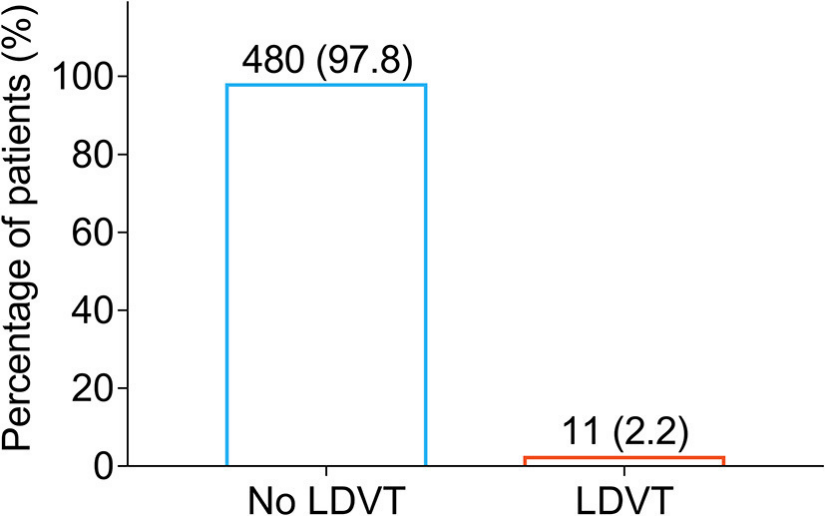
Percentage of LDVT patients in breast cancer patients who underwent surgical resection. LDVT, low-extremity deep vein thrombosis.

Low-extremity deep vein thrombosis was examined on the day of discharge from the hospital and 1 month after the discharge. Among 491 patients with breast cancer who underwent surgical resection, there were only 11 (2.2%) patients who occurred LDVT and 480 (97.8%) patients who did not occur LDVT (Figure 1). Meanwhile, the incidence of venous thromboembolism (VTE) in cancer patients from previous studies (10, 26, 30–34) is listed in Supplementary Table S1.

### Correlations Between Clinical Characteristics and LDVT

In terms of demographic characteristics and medical history, higher age (*p* = 0.002) and history of diabetes mellitus (*p* = 0.011) were correlated with increased LDVT incidence (Table 2). With respect to tumor characteristics, higher T stage (*p* = 0.038) and advanced TNM stage (*p* = 0.040) were correlated with increased LDVT incidence (Table 3). Concerning the blood routine/blood lipid indexes, higher platelet count (*p* = 0.038) was correlated with higher LDVT incidence (Table 4). Regarding the blood coagulation indexes, higher APTT (*p* = 0.001) was correlated with lower LDVT incidence (Table 5).

**Table 2:** Correlation between demographic characteristics/medical history and LDVT.

**Items**	**LDVT, No. (%)**		***P*-value**
	**No**	**Yes**	
Age (years), mean ± SD	52.4 ± 12.2	64.1 ± 9.0	0.002
BMI (kg/m^2^), mean ± SD	22.8 ± 2.3	23.6 ± 2.2	0.245
History of smoking			0.423
No	419 (87.3)	11 (100.0)	
Yes	61 (12.7)	0 (0.0)	
History of drinking			0.606
No	384 (80.0)	10 (90.9)	
Yes	96 (20.0)	1 (9.1)	
History of hypertension			0.500
No	330 (68.8)	6 (54.5)	
Yes	150 (31.2)	5 (45.5)	
History of hypercholesteremia			0.402
No	414 (86.3)	8 (72.7)	
Yes	66 (13.7)	3 (27.3)	
History of diabetes mellitus			0.011
No	437 (91.0)	7 (63.6)	
Yes	43 (9.0)	4 (36.4)	

*LDVT, low-extremity deep vein thrombosis; SD, standard deviation; BMI, body mass indexes*.

**Table 3:** Correlation between tumor characteristics and LDVT.

**Items**	**LDVT, No. (%)**		***P*-value**
	**No**	**Yes**	
Pathological grade			0.382
Grade 1	97 (20.2)	2 (18.2)	
Grade 2	281 (58.5)	5 (45.4)	
Grade 3	102 (21.3)	4 (36.4)	
Molecular subtype			0.830
Luminal-A	183 (38.1)	4 (36.4)	
Luminal-B	127 (26.5)	3 (27.3)	
HER2^+^	82 (17.1)	1 (9.1)	
Basal-like	88 (18.3)	3 (27.3)	
T stage			0.038
T1	105 (21.9)	0 (0.0)	
T2	315 (65.6)	8 (72.7)	
T3	60 (12.5)	3 (27.3)	
N stage			0.151
N0	272 (56.7)	4 (36.4)	
N1	113 (23.5)	3 (27.3)	
N2	85 (17.7)	4 (36.4)	
N3	10 (2.1)	0 (0.0)	
TNM stage			0.040
I	69 (14.4)	0 (0.0)	
II	304 (63.3)	6 (54.5)	
III	107 (22.3)	5 (45.5)	

*LDVT, low-extremity deep vein thrombosis; HER2^+^, Human Epidermal Growth Factor Receptor 2-positive; TNM, tumor, node, and metastasis*.

**Table 4:** Correlation between blood routine/blood lipid indexes and LDVT.

**Items**	**LDVT, No. (%)**		***P*-value**
	**No**	**Yes**	
WBC (x10^9^/L), median (IQR)	6.6 (5.3–7.6)	7.5 (6.5–7.9)	0.108
Hemoglobin (g/dL), median (IQR)	128.6 (118.4–137.3)	127.1 (109.2–133.1)	0.553
Platelet count (x10^9^/L), median (IQR)	237.0 (191.2–272.0)	253.4 (244.0–319.3)	0.038
TG (mmol/L), median (IQR)	1.2 (1.0–1.7)	1.2 (1.0–1.6)	0.838
TC (mmol/L), median (IQR)	4.6 (3.9–5.2)	4.7 (4.2–5.6)	0.241
LDL-C (mmol/L), median (IQR)	2.8 (2.4–3.2)	3.2 (2.5–3.8)	0.133
HDL-C (mmol/L), median (IQR)	1.5 (1.1–1.8)	1.3 (1.2–1.5)	0.433

*LDVT, low-extremity deep vein thrombosis; WBC, white blood cell; IQR, interquartile range; TG, triglyceride; TC, total cholesterol; LDL-C, low-density lipoprotein cholesterol; HDL-C, high-density lipoprotein cholesterol*.

**Table 5:** Correlation between blood coagulation indexes and LDVT.

**Items**	**LDVT, No. (%)**		***P-*value**
	**No**	**Yes**	
PT (sec), median (IQR)	11.2 (9.9–12.7)	10.3 (9.2–11.8)	0.142
APTT (sec), median (IQR)	28.6 (27.1–30.2)	26.5 (24.9–27.8)	0.001
TT (sec), median (IQR)	16.4 (15.4–17.6)	15.9 (15.3–16.2)	0.103
FIB (g/L), median (IQR)	2.9 (2.5–3.4)	3.3 (3.0–3.5)	0.080

*LDVT, low-extremity deep vein thrombosis; PT, prothrombin time; Sec, second; IQR, interquartile range; APTT, activated partial thromboplastin time; TT, thrombin time; FIB, fibrinogen*.

### Factors Related to the LDVT Incidence

**Figure 2:**
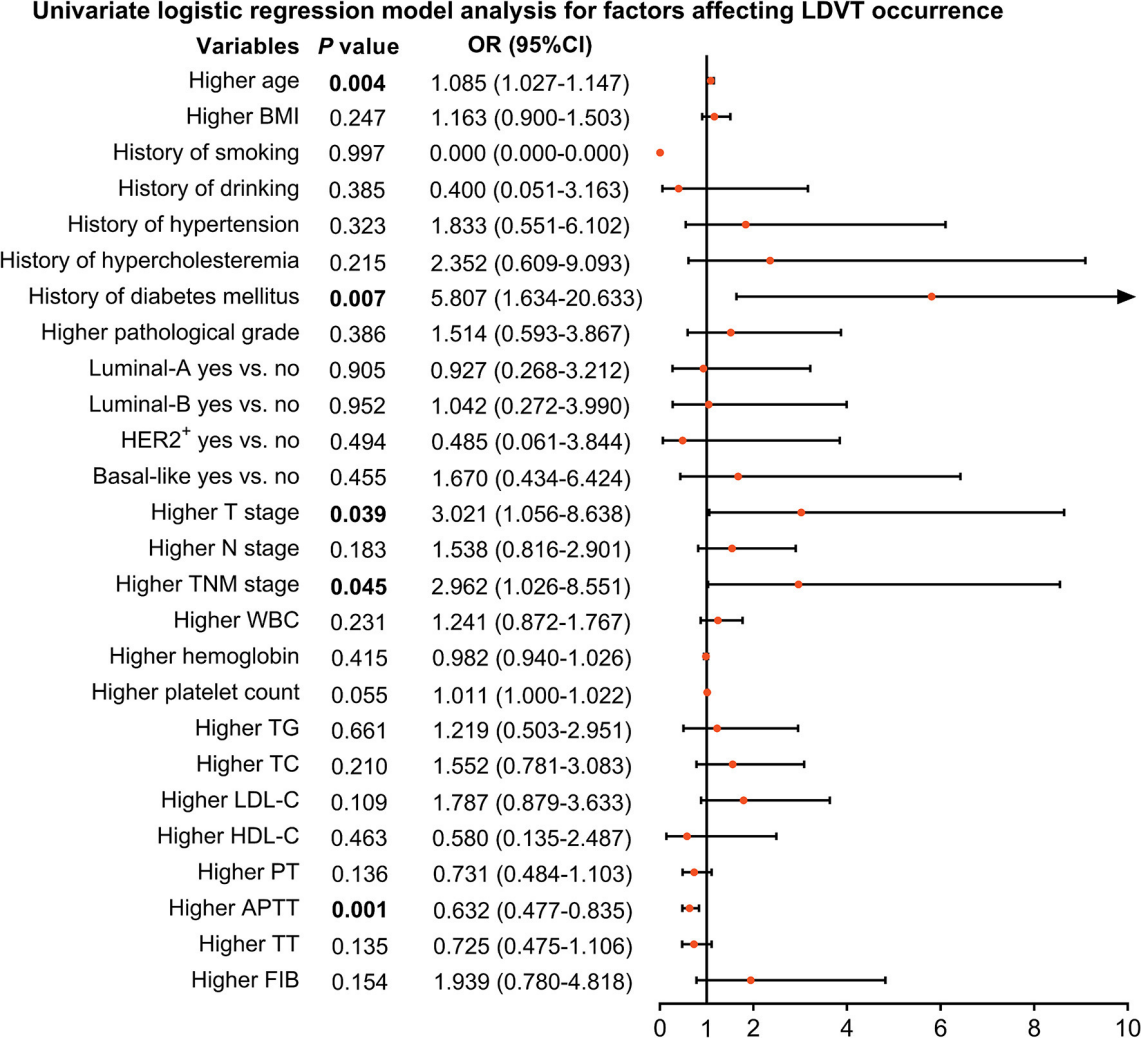
Factors impacting LDVT incidence in patients with breast cancer who underwent surgical resection. LDVT, low-extremity deep vein thrombosis; OR, odds ratio; CI, confidence interval; BMI, body mass indexes; HER2^+^, human epidermal growth factor receptor 2-positive; TNM, tumor, node, and metastasis; WBC, white blood cell; TG, triglyceride; TC, total cholesterol; LDL-C, low-density lipoprotein cholesterol; HDL-C, high-density lipoprotein cholesterol; PT, prothrombin time; Sec, second; APTT, activated partial thromboplastin time; TT, thrombin time; FIB, fibrinogen.

From the univariate logistic regression model analysis, higher age [*p* = 0.004, OR (95% CI): 1.085 (1.027–1.147)], T stage [*p* = 0.039, OR (95% CI): 3.021 (1.056–8.638)], TNM stage [*p* = 0.045, OR (95% CI): 2.962 (1.026–8.551)], and history of diabetes mellitus [*p* = 0.007, OR (95% CI): 5.807 (1.634–20.633)] were correlated with increased LDVT incidence; however, higher APTT [*p* = 0.001, OR (95% CI): 0.632 (0.477–0.835)] was correlated with lower LDVT incidence in patients with breast cancer who underwent surgical resection (Figure 2).

**Figure 3:**
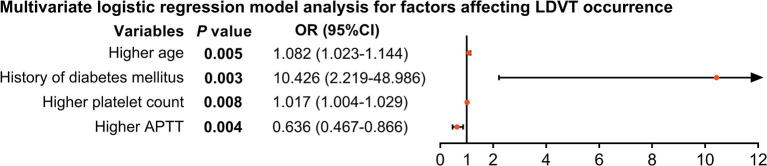
Independent factors impacting LDVT incidence in patients with breast cancer who underwent surgical resection. LDVT, low-extremity deep vein thrombosis; OR, odds ratio; CI, confidence interval; APTT, activated partial thromboplastin time.

From the step forward multivariate logistic regression model analysis, higher age [*p* = 0.004, OR (95% CI): 1.082 (1.023–1.144)], history of diabetes mellitus [*p* = 0.003, OR (95% CI): 10.426 (2.219–48.986)], and higher platelet count [*p* = 0.008, OR (95% CI): 1.017 (1.004–1.029)] were independent factors for increased LDVT incidence; whereas, higher APTT [*p* = 0.004, OR (95% CI): 0.636 (0.467–0.866)] was an independent factor for decreased LDVT incidence (Figure 3).

### Establishment of an LDVT Risk Prediction Model

**Figure 4:**
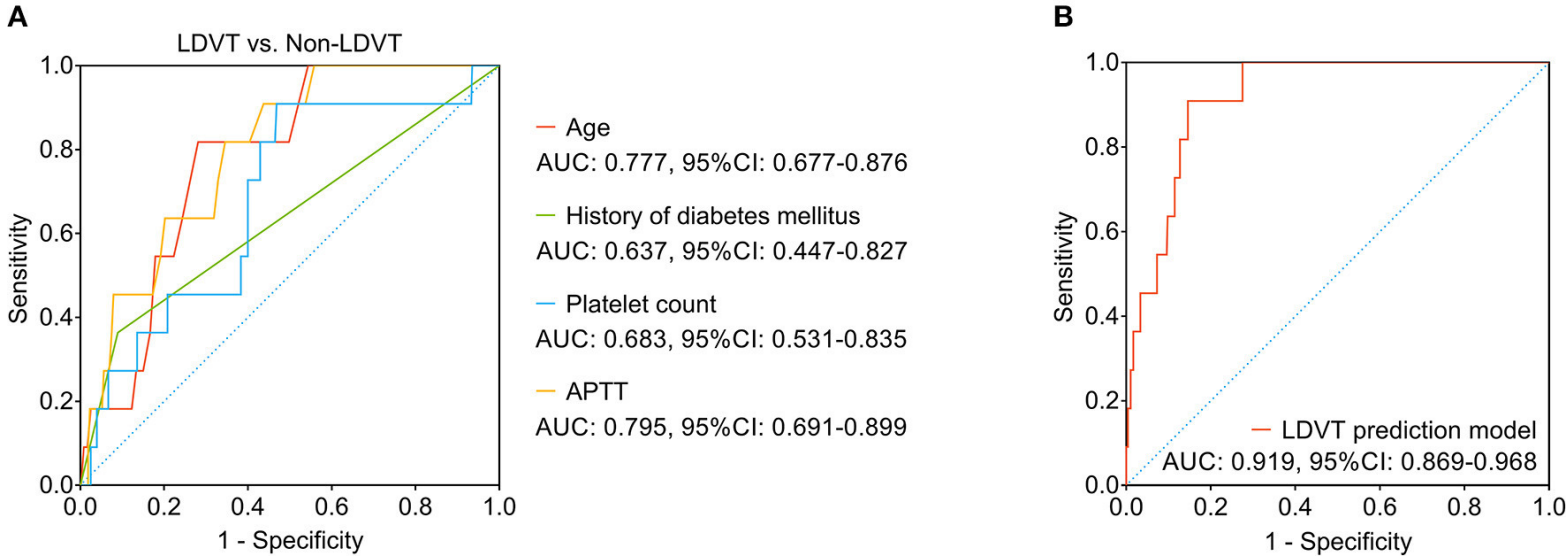
The LDVT prediction model. LDVT, low-extremity deep vein thrombosis; AUC, area under curve; CI, confidence interval. The ability of age, history of diabetes mellitus, platelet count, APTT **(A)** and the combination of these four factors **(B)** in predicting LDVT risk.

Among the abovementioned independent factors, age (area under curve (AUC): 0.777, 95% CI: 0.677–0.876) and APTT (AUC: 0.795, 95% CI: 0.691–0.899) possessed acceptable values in discriminating LDVT patients from non-LDVT patients, while the history of diabetes mellitus (AUC: 0.637, 95% CI: 0.447–0.827) and platelet count (AUC: 0.683, 95% CI: 0.531–0.835) did not possess this value (Figure 4A). It was worth noting that the combination of these four independent factors possessed a good ability to predict LDVT risk (AUC: 0.919, 95% CI: 0.869–0.968) (Figure 4B). Meanwhile, the Khorana score and the CONKO score (35) could not discriminate LDVT patients from non-LDVT patients with an AUC of 0.577 (95% CI: 0.392–0.762; Supplementary Figure S1).

## Discussion

The primary findings were listed as follows: in 491 patients with breast cancer who underwent surgical resection, (i) the incidence of LDVT was 2.2%, (ii) higher age, history of diabetes mellitus, and higher platelet count were independent factors for higher LDVT incidence, while higher APTT was an independent factor for lower LDVT incidence, (iii) the risk prediction model involving age, history of diabetes mellitus, platelet count, and APTT showed a good ability to predict LDVT occurrence.

According to previous studies, DVT rarely occurs in patients with various cancers, such as breast, prostate, and colorectal cancers (10, 18, 26, 30, 36). A study shows that in 9,735 patients with cancer (such as colorectal, lung, stomach, pancreatic, breast, and gynecologic cancers), the DVT incidence is 5.2% in Japan (37). In terms of patients with breast cancer who underwent surgery, another study exhibits that the 2-month cumulative DVT incidence is ~0.15% in the USA (26). However, limited studies estimated the LDVT incidence in patients with breast cancer. In this study, the 1-month incidence of LDVT was 2.2% (11 cases) in 491 patients with breast cancer who underwent surgical resection, which was within the range of previous studies. It could be explained by that the conditions of medical care varied in different countries, resulting in the different LDVT incidences in breast cancer.

With respect to the demographic and tumor features, they are related to DVT in patients with cancer (18, 30, 38–41). For instance, older age, obesity, and hyperlipidemia are independent risk factors for DVT in surgical colorectal cancer patients (18, 30). Moreover, the preoperative Frankel score, blood transfusion, Charlson comorbidity index, and operative time are four independent risk factors for DVT in patients with postoperative breast and cervical cancer (41). Partly in line with these reports, the current study discovered that in patients with breast cancer who underwent surgical resection, higher age, history of diabetes mellitus, T stage, and TNM stage were correlated with a higher risk of LDVT occurrence, among which higher age and history of diabetes mellitus were independent risk factors for LDVT incidence. The explanations might be as follows: (1) with the increase of age, venous thrombosis became more aggressive due to a more severe vasculopathy (17, 18), bringing in a high risk of LDVT in these patients. (2) Diabetes mellitus caused vasculopathy, neuropathy, and insufficient blood supply for extremities (42–44), enhancing the risk of LDVT in these patients. Moreover, other factors might influence the risk of LDVT, such as the application of operative mechanical pumping, which could be investigated in further studies.

Regarding the blood routine, lipid, and coagulation indexes, the present study discovered that in patients with breast cancer who underwent surgical resection, higher platelet count was an independent factor for higher LDVT incidence, while higher APTT was an independent factor for lower LDVT incidence within 1 month after discharge from the hospital. This could be attributed to the (1) higher platelet count that exerted a strong coagulation effect to form the thrombus more easily (45, 46), thus patients were at a high risk of LDVT. (2) The higher APTT indicated poor thromboplastin activity and weak coagulation effect (47, 48), which lowered the risk of thrombose and LDVT in patients. Previous studies have also suggested platelet count as a predictor of VTE in patients with cancer (49, 50); while APTT is not commonly reported (51).

To further prevent and early predict LDVT and minimize the consequence of LDVT in surgical breast cancer patients, the current study used univariate and multivariate logistic regression analyses to construct the LDVT prediction model, which discovered that the risk prediction model involves age, history of diabetes mellitus, platelet count, and APTT showed a good ability to predict LDVT occurrence. This provided evidence for the prevention and surveillance of LDVT in surgical breast cancer. Besides, previous studies also mention several risk factors for VTE, such as individual patient risk factors (such as age, sex, race, and comorbidities), cancer-associated risk factors (such as the site of cancer and the stage of cancer), and cancer-treatment-associated risk factors (such as surgery, hospitalization, and chemotherapy) (52). Some of these factors have already been included in the present study, such as age, comorbidities, and the stage of cancer. Meanwhile, considering that all the patients enrolled in this study were female breast cancer patients who underwent surgery, some of the abovementioned risk factors were not applicable in this study. Immobility is also considered as a risk factor for VTE in cancer patients (33). In the current study, we enrolled early-stage operable breast cancer patients, and the performance status was relatively satisfactory. Thus, immobility was quite rare in the current study. Several scores have been established to predict the VTE risk in patients with cancer, such as the Khorana score and the CONKO score (35). However, these scores are mainly applied in cancer patients who receive chemotherapy, while rare predictive models were established for LDVT risk in patients with breast cancer receiving surgery. Moreover, it is also suggested that chemotherapy is a considerable risk factor for VTE in patients with cancer (33, 35, 52); in the current study, all patients received surgical resection, and the administration of adjuvant therapy was given after their recoveries from the surgery, which would be 3–6 weeks after surgery, while the evaluation time of LDVT was within 1 month after surgery. Therefore, adjuvant therapy is not likely to affect the incidence of LDVT in the current study.

In spite of these findings, there were still a few limitations in this study. (1) The LDVT incidence was relatively low, so it is inadequate to evaluate the incidence and the related factors of LDVT in 491 patients with breast cancer who underwent surgical resection. (2) The current study evaluated risk factors for LDVT in breast cancer patients after resection within only a month after discharge from the hospital. Further study with a long-term follow-up was supposed to be conducted. (3) This study only evaluated the LDVT incidence and its related factors, while pulmonary embolism (PE) was also an important part of DVT. Further study should focus on the PE incidence and its related factors in surgical breast cancer patients. (4) Further studies conducted in multiple centers were needed to verify our findings.

## Conclusion

This study reveals that the LDVT incidence is 2.2%, and its independent factors consist of age, history of diabetes mellitus, platelet count, and APTT in breast cancer patients who underwent surgical resection. Although previous studies have constructed several scores to predict the risk of VTE in cancer patients, none of them are designed for patients with breast cancer who underwent surgery. The current study constructs a specific predictive model for LDVT risk in patients with breast cancer who underwent surgical resection, which provides evidence for the prevention and surveillance of LDVT in surgical breast cancer. Moreover, diabetes mellitus and APTT are recognized as risk factors for LDVT, which are seldom reported.

## Data Availability Statement

The original contributions presented in the study are included in the article/Supplementary Material, further inquiries can be directed to the corresponding author.

## Ethics Statement

The studies involving human participants were reviewed and approved by Institutional Review Board of the Fourth Hospital of Hebei Medical University. The patients/participants provided their written informed consent to participate in this study.

## Author Contributions

LL designed the study and wrote the manuscript. LC and QF performed the experiments and collected the data. WW analyzed and interpreted the data. LL and LC confirmed the authenticity of all the raw data. All authors have read and approved the final manuscript.

## Funding

This study was supported by the Medical Science Research Key Project of Hebei Province, China (no. 20180535).

## Conflict of Interest

The authors declare that the research was conducted in the absence of any commercial or financial relationships that could be construed as a potential conflict of interest.

## Publisher's Note

All claims expressed in this article are solely those of the authors and do not necessarily represent those of their affiliated organizations, or those of the publisher, the editors and the reviewers. Any product that may be evaluated in this article, or claim that may be made by its manufacturer, is not guaranteed or endorsed by the publisher.

## Supplementary Material

Supplementary Figure 1 Prediction of LDVT risk by the Khorana score and the CONKO score. LDVT, low-extremity deep vein thrombosis; AUC, area under curve; CI, confidence interval.
