# Supplementary material for: Incidence and Related Factors for Low-Extremity Deep Vein Thrombosis in Breast Cancer Patients Who Underwent Surgical Resection: What Do We Know and What Should We Care
Source: Front Surg. 2022 Feb 4;9:755671. doi: 10.3389/fsurg.2022.755671 (PMC8855971; doi:10.3389/fsurg.2022.755671)
Supplement: Supplementary Figure 1 — Prediction of LDVT risk by the Khorana score and the CONKO score. LDVT, low-extremity deep vein thrombosis; AUC, area under curve; CI, confidence interval. [file Data_Sheet_1.PDF]

Supplementary Material

Supplementary Table 1. Incidence of venous thromboembolism in cancer patients from previous studies

Study	Type of cancer	Treatment	Sample size	VTE incidence
Sharif-Kashani B (10)	Breast	Chemotherapy	403	1 (0.2%)
Andtbacka RH (26)	Breast	Surgery	4,416 procedures	7 (0.16%)
Yamashita S (30)	Colorectal cancer	Surgery	228	2 (0.9%)
Walker AJ (31)	Breast	Surgery	10,109	507 (5.0%)
	Breast	Chemotherapy	2,773	189 (6.8%)
Momeni A (32)	Breast	Surgery	52,547	395 (0.8%)
Agnelli G (33)	Various	Surgery	2,360	50 (2.1%)
Kirwan CC (34)	Breast	Chemotherapy	134	13 (9.7%)

VTE: venous thromboembolism

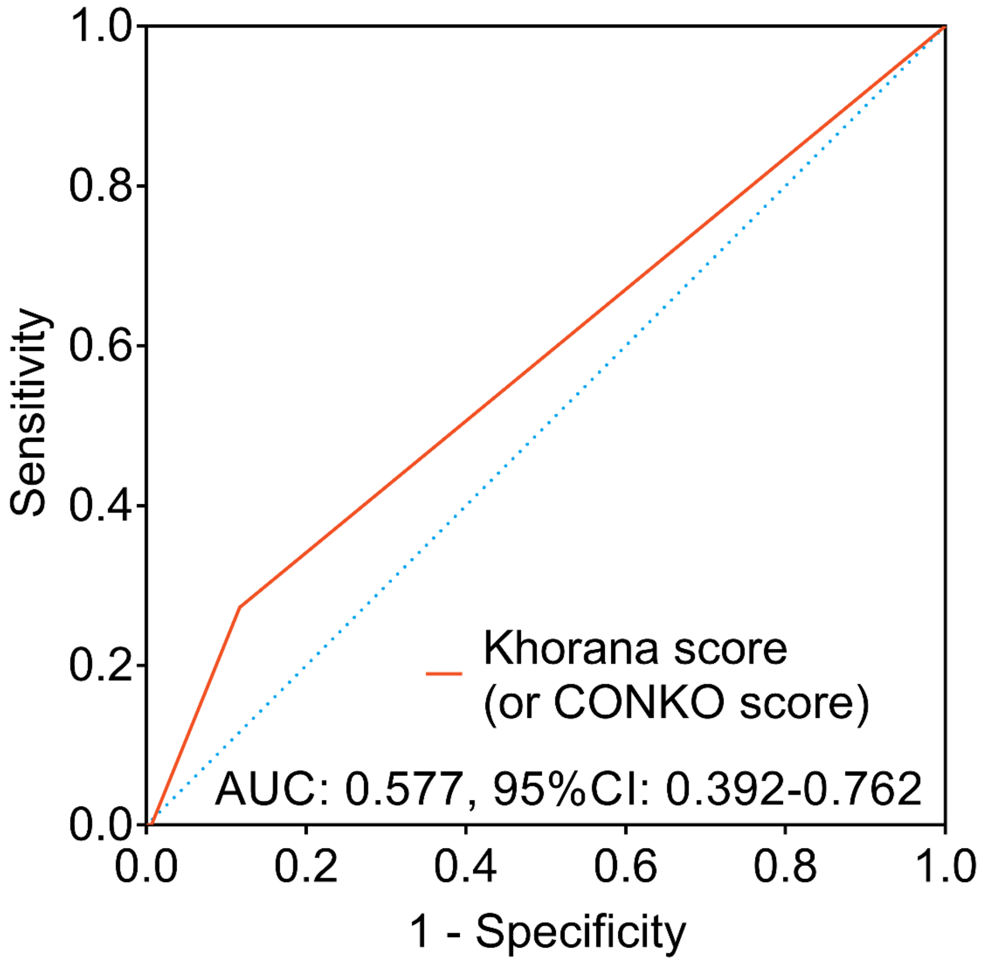

Supplementary Figure 1. Prediction of LDVT risk by Khorana score and CONKO score. LDVT, low-extremity deep vein thrombosis; AUC, area under the curve; CI, confidence interval.
